# Psychopathology and functional impairment among patients attending an adolescent health clinic: Implications for healthcare model reform

**DOI:** 10.1186/1750-4732-2-3

**Published:** 2008-02-08

**Authors:** Sushila Russell, Balakrishnan Subramanian, Paul Swamidhas Sudhakar Russell

**Affiliations:** 1 Child and Adolescent Psychiatry Unit, Department of Psychiatry, Christian Medical College, Vellore 632 002, India; 2 Department of Clinical Psychology, Sri Ramachandra Medical College and Research Institute, Chennai 600 116, India

## Abstract

**Background:**

In developing countries, primary health care facilities, such as adolescent health clinics, are frequently the first contact for an adolescent with a health professional for a myriad of health problems including mental health issues. Psychopathology is prevalent among adolescents, and causes significant educational, occupational and social impairment. The presence of psychopathology with impairment requires the development of treatment models to address both of these components. We studied the psychopathology and associated impairment in patients at an adolescent health clinic as an indicator for healthcare model reform.

**Methods:**

Psychopathology and functional impairment were assessed in 100 patients at an adolescent health clinic in the city of Chennai, Southern India. The patients had initially visited the clinic for various medical disorders. Adolescents were diagnostically classified for psychopathology using the Child Behaviour Checklist (CBCL) and the International Classification of Disease: 10^th ^Edition (ICD-10). Functional impairment was assessed with the Child Global Assessment Scale (CGAS). Data were analysed using bivariate and multivariate methods.

**Results:**

Eight percent had a diagnosable psychopathology, and they also satisfied at least one ICD-10 diagnosis. Adolescents screened had significant impairment as indicated by low CGAS scores, whether or not they presented with psychopathology. Adolescents with psychopathology were more functionally impaired both in the bivariate (Z = -3.1; P = 0.002) and multivariate analyses (β(SE) = 1.09(0.3), t = 3.9, 95% confidence interval = 0.5, 1.6; P = 0.001). Impairment in adolescents without psychopathology is primarily attributed to the medical disorders they presented with.

**Conclusion:**

Patients attending adolescent health clinics should be screened for psychopathology and functional impairment. Documented psychopathology and impairment necessitates the use of a combined treatment model to address the short and long-term problems these adolescents face.

## Background

Worldwide, one in every five people is an adolescent, (about 1.2 billion of the world's 6.3 billion people) and today, we have the largest generation of adolescents in the history of mankind [1]. There is a high prevalence of psychopathology among this population [2]. Psychopathology in adolescents has the potential to result in considerable functional impairment, frequently persists into adulthood, and can generate a large social burden if not identified and treated early. Despite these intimidating numbers and burden related to morbidity, the mental health needs of adolescents are ignored largely because the available services are inadequate [3].

A neglected, yet promising, population for early identification of psychopathology are patients attending adolescent health clinics, as mental health problems have been found to be prevalent among people in this group [4]. Targeting this population should be rewarding both in developing and industrialised countries for different reasons. In developing countries, primary health care facilities like adolescent health clinics are often the first step in the pathway to mental health, and are sometimes the only contact an adolescent has with a health professional to address a myriad of mental health problems. In industrialised countries, primary care paediatricians often handle the initial management of these adolescents with mental health needs [5], yet more effective treatment models are just as essential in these countries.

Among adolescents attending a health clinic, only 20 percent receive proper mental health service, whereas 80 percent get appropriate medical service [6]. Because of the large numbers of adolescents with mental health problems who seek care at health clinics, this setting provides a natural environment in which to develop more efficient mental health treatment models that address the barriers to adolescent mental health services such as early identification and management of the primary mental health disorder [7]. One of the deficits in existing treatment models could be the traditional dichotomy that treats clinically identified disorders using a clinical service model and that treats impairments with a population health model [8]. Despite increased awareness of the existence of psychopathology among adolescents, relatively few studies have been undertaken to document the psychopathology and impairment in adolescent health clinic settings in an effort to formulate treatment models.

The aim of the study is to document the psychopathology and the consequent impairment among adolescents attending an adolescent health clinic, and to emphasise the need for a comprehensive treatment model to address both the disorder and the associated impairment.

## Methods

### Setting and sample

This study was conducted at the adolescent health clinic located at the Sri Ramachandra Medical College and Research Institution in Southern India between January and June 2002. This teaching hospital clinic provides medical services to adolescents who reside in the city of Chennai (Madras). Adolescents attend the clinic by appointment or drop-in.

Any adolescents who met the selection criteria were included in the study. To be included in the study, adolescents needed to be between 12 and 18 years of age and enrolled in the adolescent health clinic for any general medical illness. The exclusion criteria were the presence of mental retardation or organic brain syndromes (ruled out clinically), the adolescent not being accompanied by at least one primary caregiver, and the lack of working knowledge of English or Tamil. A trained postgraduate level clinical psychologist approached the adolescents and their primary caregiver to participate in the study according to the protocol approved by the hospital's institutional review board. Written informed consent from the primary caregiver as well as verbal assent from the adolescent was obtained before data collection, and no qualified subject declined to participate in the study.

Adolescents who satisfied the selection criteria (N = 100) were interviewed and assessed using the following clinical diagnostic criteria and psychometrically sound measures.

### Measures

The *Child Behaviour Checklist *(CBCL), one of the widely used measures of childhood psychopathology, is a 118-item inventory. CBCL gives a profile composed of nine problem scale scores, two broadband scale scores and the total problems score [9]. The *Children's Global Assessment Scale *(CGAS) is a 100-point scale in which 1 indicates the most severe impairment and 100 indicates the highest level of functioning [10]. The global assessment takes into account the observations done in the functional domains of home, school and interactions with peers. Based on the total CGAS score, the adolescents in this study were classified as superior functioning (100-91), good functioning (90-81), no more than a slight impairment in functioning (80-71), some difficulty in a single area, but generally functioning pretty well (70-61), variable functioning with sporadic difficulties (60-51), moderate degree of interference in functioning (50-41), major impairment to functioning in several areas (40-31), unable to function in almost all areas (30-21), needs considerable supervision (20-11), and needs constant supervision (10-1)). The *Children's Global Assessment Scale *has been validated in many developing countries for the measurement of impairment, and is of heuristic value to complement other methods of diagnostic categorization [11]. A trained postgraduate level clinical psychologist collected details on psychopathology and functional impairment in the adolescent from the accompanying caregiver using the CBCL (parent version) and the CGAS respectively. Furthermore, those classified with psychopathology in the CBCL were screened with the *International Classification of Disease: 10th Edition *(ICD-10) [12] to see if they would satisfy any clinical diagnosis. Using the clinical interview of the adolescent and the CBCL details from the primary caregiver, the ICD-10 diagnosis was extrapolated. Based on its proven international, standard diagnostic classification utility that is widely used in general practice and in India, the ICD-10 was used for screening psychopathology instead of the ICD-9-CM or the DSM-IV-TR [13].

### Data analysis

The CBCL score (T) of 50 for both genders [9] was used to divide the sample in to adolescents with and without psychopathology. Those with a score above 50 were considered to have psychopathology and those with a score below 50 were not. Also, CGAS scores of below 71 and 61 were used to define adolescents with probable and definite functional impairment respectively [11] Comparison between groups was done with Mann-Whitney U test or Chi square test with Yates' correction. As none of the factors were significantly different between groups in the bivariate analysis, only factors shown in earlier studies to be related to impairment such as age, gender, and socio-economic status, were controlled for their potential confounding effect on the psychopathology and impairment of functioning with multiple linear regression analysis. A *post hoc *power calculation was done on the difference in the mean (sd) CGAS score between the psychopathology and no psychopathology groups [10.1(2.7)]. With a sample size of 100 participants, the study had 90 percent power to detect a clinically meaningful CGAS score difference of 10–20, which also forms the difference between two CGAS clinical scoring categories. Significance was set at P < 0.05, two tailed (SPSS 10.0).

## Results

Fifty-one males and 49 females with a mean (sd) age of 14.7 years (SD = 1.3; range = 13–18) participated in the study. The mean (sd) CBCL was 26.7(14.8) for the entire population. There was 1 adolescent with variable functioning with sporadic difficulties, 6 participants with moderate degree of interference in functioning, 23 with major impairment of functioning in several areas, 56 were unable to function in almost all areas, and 14 adolescents needed considerable supervision. There was no statistically significant difference in the age [14.7(1.3) vs.14.3 (1.5) years, Z = -0.8; P = 0.4)] between the group without psychopathology and the group with psychopathology. Also, the gender (χ^2 ^= 0.50, df = 1, P = 0.4), residence (χ^2 ^= 3.9, df = 2, P = 0.1), socio-economic status (χ^2 ^= 0.40, df = 1, P = 0.5), family structure (χ^2 ^= 0.40, df = 1, P = 0.5), level of literacy (χ^2 ^= 5.9, df = 7, P = 0.5), status of employment among adolescents (χ^2 ^= 0.30, df = 1, P = 0.6) and parental psychopathology (χ^2 ^= 0.01, df = 1, P = 0.9) were not statistically significant between groups.

Eight adolescents had a CBCL score of 50 or more and were diagnostically categorised as psychopathology cases. These adolescents had ICD-10 diagnoses of mood disorder (n = 3), anxiety disorder (n = 2), dissociative disorder (n = 2), and attention deficit with hyperactivity disorder (n = 1). All adolescents with psychopathology and without psychopathology had CGAS scores below 61, and therefore had definite functional impairment in one or more areas. Adolescents in the psychopathology group were unable to function in all three areas of home, school and in interactions with peers (CGAS score of 21–30: unable to function in almost all areas), whereas in the group without psychopathology, the majority of the adolescents had major impairment in functioning in several areas, but were unable to function in only one of the three areas (CGAS score of 31–40: major impairment to functioning in several areas). Many of those adolescents without psychopathology were otherwise functioning better with a moderate degree of interference in functioning in most social areas, or with a severe impairment of functioning in one of the areas, (CGAS score of 41–50: moderate degree of interference in functioning) but were never totally incapacitated in any of the three areas. The difference in the severity of impairment of functioning was significantly higher among adolescents with psychopathology than in those without psychopathology in the bivariate analysis as demonstrated by their global CGAS scores [30.1 (7.3) vs. 40. 2 (10.0), Z = -3.1; 95% confidence interval = 6.9 to 13.3, P = 0.002]. The CGAS score continued to be significantly lower (β(SE) = 1.09(0.3), t = 3.9, 95% confidence interval = 0.5 to 1.6; P = 0.001) among adolescents with psychopathology than in adolescents without psychopathology, even after adjusting for the confounding variables with multiple regression analysis.

## Discussion

Our result documents that 8 percent of the population attending an adolescent health clinic has a diagnosable psychopathology, which is only marginally lower than the 9.5 percent and 12.5 percent prevalence rate reported by the British and Indian community surveys respectively [14,15]. This demonstrates that the prevalence of psychopathology among adolescents attending adolescent health clinics is as high as in the general populations. More significant is the finding that all adolescents who participated in the study were significantly functionally impaired whether they had a diagnosable psychopathology or not. However, the adolescents with psychiatric disorders had significantly more impairment than those without psychopathology. The presence of functional impairment in children without psychopathology is explained by the fact that these adolescents had medical disorders, [16] and also by the possibility that they might have had subclinical psychiatric morbidity [17] further compromising their functioning. This prevalence of psychiatric disorders with significant impairment raises reservations about the psychiatric treatment models available for the adolescents attending these clinics.

The goal of children's mental health programs is the sound development and well-being of all children, with a reduction of impairment caused by psychiatric disorders [18]. It is, therefore, essential to address the symptoms, impairment and burden caused by these disorders [19,20]. Traditionally, symptoms and impairment caused by disorders have been managed with different health care models such as the clinical service model and the population health model respectively [8]. In this dichotomised model, the population health model focuses on enhancing family incomes, social supports, early child development, and other nonmedical determinants of health for whole populations or for subpopulations of children, and therefore, only addresses the impairment in these areas [21,22]. On the other hand, clinical service models often exclusively provide only diagnostic and treatment services for individuals who have disorders, despite the fact that these two approaches can complement each other to improve health outcomes [23]. To resolve mental health problems seen in adolescent health clinics, combining both the population health model and the clinical service model is essential to reducing the significant symptomatology, the impairment, the propensity for problems to continue into adulthood, and the burden to society that is often seen in these cases [24]. This combined model should be advocated as one of the mainstream models for diagnosing and treating psychiatric disorders among adolescents attending adolescent health clinics, in contrast to the dichotomised model currently used.

Combining these models can narrow the dissonance between symtomatology and impairment, as symptoms alone do not equate to the individual's needs. It should also be noted that many people who meet diagnostic criteria for a mental disorder function effectively. Policies allocating treatment resources equally to inpatient and outpatient care, instead of the current practice of devotion to inpatient care, will improve the resources for outpatient services like the adolescent health services clinics to focus on specialised mental health care.

The caveat of this study is that the children with psychopathology also had medical disorders that were not categorised. The categorisation of different medical disorders, with a potential to result in impairment, and adjustment for their confounding effect, would have further supported the impairment caused by the psychopathology demonstrated in this study. Also, we conducted a *post hoc *power calculation that reportedly has shortcomings when compared with an *a priori *sample size calculation [25]. Although confidence interval was mentioned where appropriate to overcome this limitation [26], the lack of an *a priori *sample size calculation might have compromised the power of the study. Finally, the findings are not based on a multicentric study and therefore the generalisability of the findings might be limited.

In conclusion, despite these limitations, this study demonstrates the prevalence of psychopathology in an adolescent clinic population to be as high as in the general population. These adolescents also have been documented to have significant functional impairment. Therefore, the existing models available to address the mental health of adolescents seeking care at adolescent health clinics need to be reviewed and should address both the disorder and the impairment with holistic treatment models. Future study designs in adolescent mental health care in primary care settings should be longitudinal and multicentric in nature to avoid the effect of seasonality on the prevalence of psychopathology and consequent impairment.

### Implications for healthcare reforms

The documentation of both psychopathology and impairment among patients attending an adolescent health clinic brings to light some of the significant healthcare model changes that are required in behavioral health services as part of primary care services in resource-poor countries. Integrating the clinical service model and population health model in behavioral health services in such countries will significantly improve the quality of life among adolescents as many nonmedical determinants of health can be enhanced.

## List of abbreviations

CBCL = Child Behaviour Checklist; CGAS = Child Global Assessment Scale; DSM-IV-TR = Diagnostic and Statistical Manual of Mental Disorders, Fourth Edition, Text Revision.

ICD-9-CM = International Classification of Diseases, 9th Revision, Clinical Modification; ICD-10 = International Classification of Diseases, Clinical Guidelines Diagnostic Criteria Version, 10th Edition.

## Competing interests

The author(s) declare that they have no competing interests.

## Authors' contributions

SR was involved in the conception, drafting and revising the final draft. BS was involved in the conception, drafting and revising the final draft. PSSR was involved in conception, designing, data analysis and interpretation and approving the final version.
